# Multi-loop traction device facilitates gastric endoscopic submucosal dissection: ex vivo pilot study and an inaugural clinical experience

**DOI:** 10.1186/s12876-021-02085-w

**Published:** 2022-01-06

**Authors:** Hiroaki Matsui, Naoto Tamai, Toshiki Futakuchi, Shunsuke Kamba, Akira Dobashi, Kazuki Sumiyama

**Affiliations:** Department of Endoscopy, The Jikei University School of Medicine, 3-25-8 Nishi Shinbashi, Minato-ku, Tokyo, 105-8461 Japan

**Keywords:** Multi-loop traction device, Endoscopic submucosal dissection, Gastrointestinal tract, Tumors, Lesions

## Abstract

**Background:**

Endoscopic submucosal dissection (ESD) is technically difficult and requires considerable training. The authors have developed a multi-loop traction device (MLTD), a new traction device that offers easy attachment and detachment. We aimed to evaluate the utility of MLTD in ESD.

**Methods:**

This ex vivo pilot study was a prospective, block-randomized, comparative study of a porcine stomach model. Twenty-four lesions were assigned to a group that undertook ESD using the MLTD (M-ESD group) and a group that undertook conventional ESD (C-ESD group) to compare the speed of submucosal dissection. In addition, the data of consecutive 10 patients with eleven gastric lesions was collected using electronic medical records to clarify the inaugural clinical outcomes of gastric ESD using MLTD.

**Results:**

The median (interquartile range) speed of submucosal dissection in the M-ESD and C-ESD groups were 141.5 (60.9–177.6) mm2/min and 35.5 (20.8–52.3) mm2/min, respectively; submucosal dissection was significantly faster in the M-ESD group (p < 0.05). The rate of en bloc resection and R0 resection was 100% in both groups, and there were no perforation in either group. The MLTD attachment time was 2.5 ± 0.9 min and the MLTD extraction time was 1.0 ± 1.1 min. Clinical outcomes of MLTD in gastric ESD were almost the same as those of ex vivo pilot study.

**Conclusions:**

MLTD increased the speed of submucosal dissection in ESD and was similarly effective when used by expert and trainee endoscopists without perforation. MLTD can potentially ensure a safer and faster ESD.

**Supplementary Information:**

The online version contains supplementary material available at 10.1186/s12876-021-02085-w.

## Background

Endoscopic submucosal dissection (ESD) enables *en bloc* endoscopic resection of superficial tumors of the gastrointestinal tract, regardless of size or the presence of fibrosis in the submucosal layer [1–3]. However, ESD is technically difficult, and requires a longer procedure time than endoscopic mucosal resection (EMR) does; therefore, mastering this technique requires extensive training [4, 5]. Controlling intraoperative bleeding during ESD is difficult, and perforation is more common than with EMR. These complications are more likely to occur when there is inadequate visualization of the submucosa [6].

Various devices have been developed to improve the safety of ESD, including traction devices, which hold back the mucosa to facilitate proper visualization of the submucosa. To date, traction devices have been reported to improve the safety and efficacy of ESD; however, several difficulties when using these devices remain. Specifically, the challenges associated with traction devices include the difficulty of delivering the device to the lesion site and the potential obstruction posed by the device, because the direction of traction cannot be changed after attachment.

We developed a new traction device, called the multi-loop traction device (MLTD; Boston Scientific Co. Ltd., Tokyo, Japan), which has three connected rings made of a unique linear biocompatible low-density polyethylene plastic. The MLTD can be delivered to the lesion site from the forceps channel of an endoscope and is simple to attach. The middle ring can even be used to change the direction of traction after the attachment, and the MLTD can be easily cut and removed simply by lightly grasping one of the rings using biopsy forceps and pulling away. The objective of this study was to determine the utility of MLTD in ESD using a resected porcine stomach model.

## Methods

### The ex vivo pilot study

This was a prospective, block-randomized, comparative study that used a resected porcine stomach model (Tokyo Shibaura Zouki Co., Ltd., Tokyo, Japan) to evaluate the utility of the MLTD in ESD. Twenty-four lesions (four lesions/endoscopist among three expert endoscopists, and four lesions/endoscopist among three trainees inexperienced in clinical ESD) were assigned in a 1:1 ratio between a group that undertook ESD using the MLTD (M-ESD group) and a group that undertook conventional ESD (C-ESD group). Expert endoscopists were defined on the basis of being a Japan Gastroenterological Endoscopy Society medical specialist with > 50 instances of clinical experience in ESD. Trainees were defined as gastroenterologists with no clinical experience with ESD (Additional file 1: Table S1).

Before participation in this study, trainees undertook training consisting of performing ESD on two lesions in a resected porcine stomach model. In this study, trainees performed ESD procedures under the supervision of expert endoscopists.

### Randomization method

Lesions were assigned to endoscopists using MS Excel (Microsoft, Redmond, WA, USA) to assign a random number to each lesion and then randomly allocate to either the M-ESD group or C-ESD group. The lesion site (anterior or posterior wall) was also used as a random block factor.

### ESD procedure

ESD was performed with a distal tip attachment (D-201-10704; Olympus Co. Ltd., Tokyo, Japan) attached to a GIF-Q260 endoscope (Olympus Co. Ltd., Tokyo, Japan). All circumferential incisions and submucosal dissections were performed using DualKnife (KD-655L; Olympus Co. Ltd., Tokyo, Japan). The fluid injected into the local submucosa was a mixture of 0.4% hyaluronic acid solution (MucoUp®, Boston Scientific Co. Ltd.) and a small amount of indigo carmine. A high-frequency generator (ESG-100, Olympus Co. Ltd., Tokyo, Japan) was used for this study, which was set to the ForcedCoag mode (50 W) and PulseCut fast mode (70 W).

Before starting the ESD procedure, a 20-mm diameter disk was placed in the resected porcine stomach to mark the stomach and create a 20-mm diameter mock lesion. Next, a mixture of hyaluronic acid and a small amount of indigo carmine was injected into the submucosal layer to elevate the lesion. Up to this point, the ESD procedure was identical in the M-ESD and C-ESD groups.

**Fig. 1:**
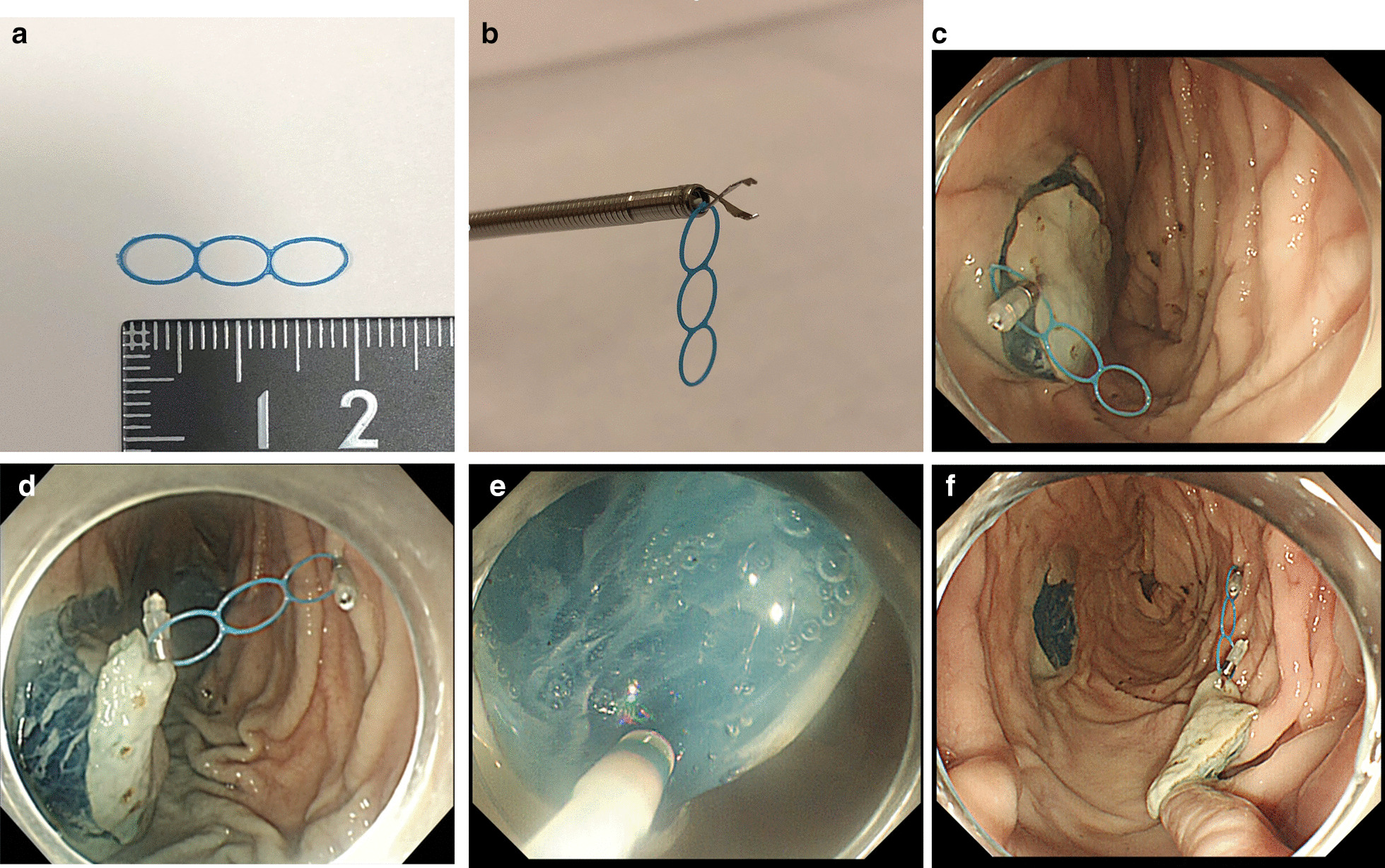
ESD procedure using MLTD. **a** Multi-loop traction device (MLTD). **b** Snag the MLTD at the base of the clip, mount the clip in the rotatable clip device, and deliver into the stomach through the forceps port. **c** Deliver a clip with MLTD attached from the end of the endoscope, and attach to the proximal side of the lesion mucosa. **d** Using another clip, snag an MLTD ring, and apply countertraction by attaching the clip to the gastric wall contralateral to the lesion. **e** Optimal traction and visualization are obtained during submucosal dissection. **f** The lesion is completely resected. ESD, endoscopic submucosal dissection; MLTD, multi-loop traction device

In the C-ESD group, dissection of the submucosal layer was performed after completion of the circumferential incision around the lesion. In the M-ESD group, the MLTD was attached between the mucosa around the lesion and the porcine gastric wall contralateral to the lesion after the circumferential incision, using a short-type clip (EZ clip, Olympus Co. Ltd., Tokyo, Japan), which was reloadable (Fig. 1a, b). First, the MLTD was snagged at the base of the clip; thereafter, the clip with the MLTD attached was delivered into the porcine stomach model through a forceps channel using a rotatable clip device (HX-201LR-135; Olympus Co. Ltd., Tokyo, Japan). The clip was then attached to the edge of the proximal side of the lesion mucosa (Fig. 1c). Another reloadable short clip was used to snag the ring on the other end of the MLTD and create countertraction by attaching it to the gastric wall contralateral to the lesion, enabling good visualization of the submucosa (Fig. 1d). Finally, submucosal dissection was performed (Fig. 1e), and the lesion was resected, identical to how it was performed in the C-ESD group (Fig. 1f).

After resection, the MLTD was retrieved together with the lesion using biopsy forceps to grasp the MLTD and pull it out. After resection of the lesion, the lesion specimen was retrieved from the porcine stomach model using an endoscope, and the size of the specimen was measured. Video recordings were performed for all endoscopy procedures (Additional file 2: Video S1).

### MLTD

The MLTD is a detachable and commercially available traction device for ESD that consists of three connected rings of a unique linear biocompatible low-density polyethylene plastic. The device is 0.3 mm thick, and each ring is 6 mm in diameter. The MLTD can be delivered to the lesion site from the forceps channel of an endoscope using a clip device. is easily cut and removed by lightly grasping the loop of the MLTD with biopsy forceps and pulling it away. MLTD is easily cut and removed by lightly grasping the loop that is attached to the gastrointestinal (GI) tract wall by the clip, with biopsy forceps and pulling it away. When the tension on the MLTD decreases or the direction of tension becomes inadequate, re-tensioning or re-direction is allowed by reattaching the middle ring of the MLTD to the GI tract wall. For clinical use, MLTD has to be retrieved from the GI tract together with the lesion or through the forceps channel using biopsy forceps after completion of ESD.

### Endpoints

The primary endpoint in this study was the submucosal dissection speed (mm^2^/min), which was compared between the M-ESD and C-ESD groups. The secondary endpoints were the rate of procedure completion, rate of *en bloc* resection, rate of R0 resection, rate of perforation, overall procedure time, injection time, perimeter incision time, submucosal dissection time, total local injection volume, resected specimen area, and number of additional local injections during submucosal dissection.

The rate of MLTD attachment, rate of successful MLTD attachment, rate of specimen retrieval with biopsy forceps, MLTD attachment time, and MLTD retrieval time were also analyzed in the M-ESD group.

In the C-ESD group, the overall procedure time was defined as the time from first submucosal injection to completion of specimen removal; in the M-ESD group, it was defined as the time from first submucosal injection to MLTD retrieval. When all markings on the mock lesion were identified in the resected and retrieved specimens, we recognized that the lesion was resected as R0. Successful MLTD attachment was defined as achieving good traction with clips attached to the lesion and the gastric wall contralateral to the lesion.

### Sample size calculation

A previous report [7] noted that the mean dissection speed of gastric ESD in living pigs without traction was 0.6 ± 0.1 cm^2^/min. Assuming that the dissection speed will be 20% faster with the MLTD, to detect a significant difference compared to C-ESD with an alpha error of 0.05, and a statistical power of 80%, each group required 12 lesions.

### Statistical analysis

All variables are presented as median and interquartile (IQR) range (first quartile—third quartile [IQR]) or median with range, with a two-sided significance level of 5%. Categorical variables were compared using the chi-squared test and Fisher’s exact test, and continuous variables were compared using the Mann–Whitney U test. A p value less than 0.05 was considered statistically significant. All analyses were performed using Stata 14.0 (StataCorp LP, College Station, TX, USA).

### Clinical feasibility evaluation of gastric ESD using MLTD

To clarify the inaugural clinical outcomes of gastric ESD using MLTD, the data of consecutive patient who received gastric ESD using MLTD from September to December 2020 at our institution was collected using electronic medical record. The gastric lesion resected by gastric ESD with partial use of MLTD for submucosal dissection was excluded from the evaluation. The study was approved by the ethics committee of the Jikei University School of Medicine, Tokyo, Japan (no. 31–109(9608)), and it conforms to the provisions of the Declaration of Helsinki.

## Results

### Primary endpoint

Submucosal dissection speed during gastric ESD was significantly faster in the M-ESD group than in the C-ESD group (141.5 [60.9–177.6] vs. 35.5 [20.8–52.3] mm^2^/min, *p* < 0.05) (Table 1). Even when the analysis was divided between expert and trainee endoscopists, submucosal dissection speed remained significantly faster in the M-ESD group (M-ESD vs. C-ESD among experts: 168.8 [150.5–280.6] vs. 48.0 [31.1–83.6] mm^2^/min, *p* < 0.05) (M-ESD vs. C-ESD among trainees: 60.2 [56.2–73.0] vs. 23.8 [20.4–42.0] mm^2^/min, *p* < 0.05).

**Table 1:** Multi-loop traction device-assisted ESD versus conventional ESD in the ex vivo pilot study

Submucosal dissection speed (mm^2^/min); median (IQR)	M-ESD	C-ESD	*p* value
Overall (n = 24)	141.5 (60.9–177.6)	35.5 (20.8–52.3)	< 0.05
Experts only (n = 12)	168.8 (150.5–280.6)	48.0 (31.1–83.6)	< 0.05
Trainees only (n = 12)	60.2 (56.2–73.0)	23.8(20.4–42.0)	< 0.05

ESD procedure outcomes	M-ESD (n = 12)	C-ESD (n = 12)	*p* value
Rate of *en bloc* resection (%)	100 (12/12)	100 (12/12)	–
Rate of R0 resection (%)	100 (12/12)	100 (12/12)	–
Rate of accidental complications (perforation) during ESD (%)	0 (0–6)	0 (0–6)	–
Overall procedure time (min); median (IQR)	24.0 (17–40)	30.0 (19.8–48.8)	0.56
Local injection time (min); median (IQR)	3.5 (3–5)	3.5 (2.8–8.3)	0.91
Perimeter incision time (min); median (IQR)	7.5(3.8–16.5)	6.0 (3.8–7.3)	0.52
MLTD attachment time (min); median (IQR)	2.5 (2–3)	–	–
Submucosal dissection time (min); median (IQR)	7.5 (3–11.3)	20.0 (10.5–27)	< 0.05
MLTD retrieving time (min); median (IQR)	1.0 (1–1)	–	–
Total local injection volume (mL); median (IQR)	22.5 (16.5–29)	24.0 (18–34.8)	0.58
Specimen area (mm^2^); median (IQR)	854.1 (559.3–1003.2)	637.8 (559.3–847.8)	0.27
Number of additional local injections; median (IQR)	0 (0–0)	0 (0–0.3)	0.68

C-ESD, conventional ESD; ESD, Endoscopic submucosal dissection; M-ESD, Multi-loop traction device-assisted ESD; MLTD, multi-loop traction device; IQR, interquartile range

### Secondary endpoints

The rate of procedure completion, rate of *en bloc* resection, and R0 resection were both 100% in the M-ESD group and the C-ESD group. The rate of perforation was 0% in both groups. The submucosal dissection time was significantly shorter in the M-ESD group than in the C-ESD group (7.5 [3–11.3] vs. 20.0 [10.5–27] min, *p* < 0.05). There were no significant differences between the groups in terms of overall procedure time, local injection time, perimeter incision time, total local injection volume, resected specimen area, and number of additional local injections during submucosal dissection.

Even when the analysis was limited to expert endoscopists, the submucosal dissection time was significantly shorter in the M-ESD group than in the C-ESD group (3.0 [2.3–6] vs.12.5 [8.3–17.5] min, *p* < 0.05) (Table 2). When the analysis was limited to trainee endoscopists, there was no significant difference in the submucosal dissection time between the groups (Table 3).

**Table 2:** ESD procedure outcomes (expert endoscopist): multi-loop traction device-assisted ESD versus conventional ESD (n = 12) in the ex vivo pilot study

	M-ESD (n = 6)	C-ESD (n = 6)	*p* value
Rate of *en bloc* resection (%)	100 (6/6)	100 (6/6)	–
Rate of R0 resection (%)	100 (6/6)	100 (6/6)	–
Rate of accidental complications (perforation) during ESD (%)	0 (0–6)	0 (0–6)	–
Overall procedure time (min); median (IQR)	17.0 (13.3–18.5)	22.5 (14.5–29)	0.20
Local injection time (min); median (IQR)	3 (2.3–3)	3 (2.3–3)	1
Perimeter incision time (min); median (IQR)	3.5 (2.3–5.5)	4.5 (3–6)	0.68
MLTD attachment time (min); median (IQR)	2.0 (1.3–2.8)	–	–
Submucosal dissection time (min); median (IQR)	3 (2.3–6)	12.5 (8.3–17.5)	< 0.05
MLTD extraction time (min); median (IQR)	1.0 (1–1)	–	–
Total local injection volume (mL); median (IQR)	17.5 (11.3–23.8)	21.5 (14–24.5)	0.47
Specimen area (mm^2^); median (IQR)	706.5 (471–942)	647.6 (458.0–812.5)	0.42
Number of additional local injections; median (IQR)	0 (0–0)	0 (0–0)	0.32

ESD, Endoscopic submucosal dissection; MLTD, multi-loop traction device; IQR, interquartile range; C-ESD, conventional ESD; M-ESD, Multi-loop traction device-assisted ESD

**Table 3:** ESD procedure outcomes (trainee endoscopist): multi-loop traction device-assisted ESD versus conventional ESD (n = 12) in the ex vivo pilot study

	M-ESD (n = 6)	C-ESD (n = 6)	*p* value
Rate of *en bloc* resection (%)	100 (6/6)	100 (6/6)	–
Rate of R0 resection (%)	100 (6/6)	100 (6/6)	–
Rate of accidental complications (perforation) during ESD (%)	0 (0–6)	0 (0–6)	–
Overall procedure time (min); median (IQR)	43.0 (35.5–62.5)	50.5 (43.3–54)	1.00
Local injection time (min); median (IQR)	5 (4.3–8)	8.5 (7.3–9)	0.52
Perimeter incision time (min); median (IQR)	17 (12.3–19.5)	7.0 (5.3–13.3)	0.13
MLTD attachment time (min); median (IQR)	3.0 (2.3–3)	–	–
Submucosal dissection time (min); median (IQR)	12.5 (9.3–21)	28.0 (25.3–31.5)	0.08
MLTD extraction time (min); median (IQR)	1.0 (1–1.75)	–	–
Total local injection volume (mL); median (IQR)	31.0 (21.8–38.8)	36.5 (24–47.5)	0.57
Specimen area (mm^2^); median (IQR)	854.1 (714.4–1158.1)	637.8 (598.6–880.8)	0.23
Number of additional local injections; median (IQR)	0 (0–0.75)	0 (0–0.75)	0.92

ESD, Endoscopic submucosal dissection; MLTD, multi-loop traction device; IQR, interquartile range; C-ESD, conventional ESD; M-ESD, Multi-loop traction device-assisted ESD

In the M-ESD group, the rate of successful MLTD attachment (number of successful MLTD attachment/number of attempts for MLTD attachment) was 85.7%, the rate of finally successful MLTD attachment was 100%, the rate of specimen retrieval with biopsy forceps was 100%, the median MLTD attachment time was 2.5 min (range 2–3 min), and the median MLTD retrieving time was 1.0 min (range 1–1 min). Comparing between the expert and trainee endoscopists in the M-ESD group revealed no significant difference in the rate of successful MLTD attachment, rate of final successful attachment, MLTD attachment time, rate of MLTD retrieval, and MLTD extraction time (Table 4).

**Table 4:** Multi-loop traction device procedure (Expert endoscopist vs. Trainee endoscopist [n = 12]) in the ex vivo pilot study

	Expert (n = 6)	Trainee (n = 6)	*p* value	M-ESD (Total) (n = 12)
Successful MLTD attachment rate (number of successful MLTD attachment/number of attempts for MLTD attachment) (%)	100 (6/6)	75 (6/8)	0.47	85.7 (12/14)
Finally successful MLTD attachment rate (%)	100 (6/6)	100 (6/6)	*NS*	100 (12/12)
Rate of specimen retrieval (%)	100 (6/6)	100 (6/6)	*NS*	100 (12/12)
MLTD attachment time (min); median (IQR)	2.0 (1.3–2.8)	3.0 (2.3–3)	0.31	2.5 (2–3)
MLTD extraction time (min); median (IQR)	1 (1–1)	1 (1–1.8)	0.14	1.0 (1–1)

MLTD, multi-loop traction device; M-ESD, Multi-loop traction device-assisted ESD; IQR, interquartile range

### Clinical feasibility evaluation of gastric ESD using MLTD

A total of ten patient with 11 lesions were included for evaluation of clinical outcomes of gastric ESD using MLTD. Submucosal dissection speed during gastric ESD was 37.2 (29.7–59.8). The rate of procedure completion, rate of *en bloc* resection, and R0 resection were 100%. The perforation rate was 0%. The rate of successful MLTD attachment (number of successful MLTD attachment/number of attempts for MLTD attachment) was 91.7%, the rate of finally successful MLTD attachment was 100%, the rate of specimen retrieval with biopsy forceps was 100%, the median MLTD attachment time was 1 min (range 1–2 min), and the median MLTD retrieving time was 1.0 min (range 0–1 min). (Table 5.).

**Table 5:** Clinical outcomes of gastric ESD using Multi-loop traction device in Clinical feasibility evaluation

The M-ESD (10 patients 11 lesions)	
Year (y); median (range)	74 (66–87)
Gender (male: female)	9:1
Location (U/M/L)	5/5/1
Lesion position (Greater curvature/Lesser curvature/Anterior wall/Posterior wall)	4/1/2/4
Morphology (Depressed/Flat/Protruded)	5/0/6
Lesion size (mm) (longer axis); median (IQR)	12.0 (10.0–27.5)
Lesion size (mm) (shorter axis); median (IQR)	10.0 (8.0–24.0)
Submucosal dissection speed (mm^2^/min); median (IQR)	37.2 (29.7–59.8)
Rate of en bloc resection (%)	100 (11/11)
Rate of R0 resection (%)	100 (11/11)
Rate of accidental complications (perforation) during ESD (%)	0 (0/11)
Overall procedure time (min); median (IQR)	38.0 (27.0–63.5)
MLTD attachment time (min); median (IQR)	2 (1–2)
MLTD retrieving time (min); median (IQR)	1 (0–1)
Total local injection volume (mL); median (IQR)	21.5 (16.5–43.5)
Specimen area (mm^2^); median (IQR)	706.5 (580.1–1420.1)
Presence of ulceration (%)	18.2 (2/11)
successful MLTD attachment rate (number of successful MLTD attachment/number of attempts for MLTD attachment) (%)	91.7 (11/12)
Finally successful MLTD attachment rate (%)	100 (11/11)
Rate of MLTD retrieval (%)	100 (11/11)
MLTD attachment time (min); median (IQR)	2 (1–2)
MLTD extraction time (min); median (IQR)	1 (0–1)

ESD, Endoscopic submucosal dissection; M-ESD, Multi-loop traction device-assisted ESD; IQR, interquartile range; MLTD, multi-loop traction device

## Discussion

This is the first study to evaluate the effectiveness of MLTD, a commercially available and easily detachable intraluminal traction device for ESD procedures using a porcine stomach model. Although the effectiveness of handmade multi-loop traction devices using surgical sutures has been already reported as devices that potentially facilitate ESD, handmade multi-loop traction devices are not commercially available. In addition to retrieving the handmade multi-loop, the use of an ESD knife with a high-frequency generator is required [8]. This study revealed that using MLTD increased the speed of submucosal dissection not only among experts but also among trainee endoscopists; in addition, among expert endoscopists, the submucosal dissection time was shortened. This study also revealed no significant difference in either MLTD attachment time (2.5 min) or retrieval time (1.0 min) between expert and trainee endoscopists, showing that MLTD was easy to attach and detach, even for trainees.

When performing conventional ESD, the tip of the endoscope must be slipped under the lesion to visualize the submucosal layer using the transparent attachment. However, the MLTD allows a better visualization of the submucosal layer and muscularis propria without using the tip of the endoscope; therefore, MLTD allows the anticipation of an overview of the muscularis propria and facilitates safer ESD. In this study, although MLTD attachment failed on two occasions, it was eventually successful in 100% of the cases. In addition, dropped MLTDs can still be used if a type of clip that can be re-held is used. We found no evidence of insufficient traction during dissection, and there were no cases of damage caused by excessive stretching.

A systematic review of the safety and efficacy of traction in ESD including 33 published articles, 3,134 patients, and traction techniques such as anchor-guided ESD, ESD using a second endoscope, and clip-related techniques, reported that traction was associated with a significant improvement in procedure times and rate of R0 resection, and that traction also reduced the incidence of bleeding and perforation complications [9].

Traction devices are broadly divided into two types: extracorporeal traction and intraluminal traction. Extracorporeal traction devices, such as devices that use dental floss or polyester line attached to a clip [10–13], apply traction from outside the body; hence, traction is unidirectional and applied to the proximal side. Additionally, there is limited direction of traction, and delivering traction devices is time consuming and requires re-insertion of the endoscope. Magnetic anchors [14, 15] and the S–O clip [16–19] are intraluminal traction devices that do not affect control of the endoscope, and they can apply traction on the lesion independent of the endoscope. However, only the S–O clip is commercially available. In a randomized controlled trial, Nagata et al. [19] reported that the median procedure time of gastric ESD using the S–O clip was significantly shorter than that of conventional gastric ESD (29.1 vs. 52.6 min, p = 0.05). In addition, they classified the potential direction of traction into vertical, proximal, and distal, and vertical traction was selected as optimal for gastric ESD to improve the visualization of the submucosal layer. In the present study, vertical traction was also selected as optimal for gastric ESD. The gastric ESD procedure was not performed in the retroflex position to avoid interference between the endoscope and MLTD, and excessive air insufflation was avoided to prevent unintentional falling of MLTD. Hashimoto et al. [18] also reported that the mean resection time of gastric ESD using the S–O clip was significantly shorter than that of conventional ESD (47.2 ± 24.6 vs. 69.2 ± 67.1 min, *p* = 0.035). However, the time required for S–O clip attachment was 4.4 min (range: 2–15 min). Hashimoto et al. pointed out that grasping the S–O clip with the second clip through the nylon loop of the S–O clip was occasionally time consuming.

The MLTD used in the present study is also an intraluminal traction device, although it offers some unique benefits, as it does not require an ESD knife with a high-frequency generator for retrieval and can be extracted using biopsy forceps; extraction is extremely simple and does not damage the mucosa. In addition, any type of clip can be used for attachment, making it easier to attach the MLTD to the GI tract. Furthermore, although not explored in this study, because the MLTD has multiple rings that enable re-direction of tension or re-tension during the ESD procedure, if the traction force gradually weakens as the lesion is dissected, MLTD enables optimum traction direction or force maintenance.

In addition, this report also clarified the inaugural clinical outcomes of gastric ESD using MLTD. From the results of clinical outcomes of gastric ESD with MLTD, it seems feasible to apply MLTD for clinical gastric ESD.

This study had several limitations. This was an ex vivo study that did not require hemostatic intervention during clinical ESD and evaluated ESD procedures were conducted in forward position. The sample size was small and sample size calculation was conducted in assumption of 0.6cm2/min in conventional ESD, but resulted in 35.5 mm2/min. Furthermore, the ESD sites were limited to the anterior and posterior walls of the stomach. In addition, the number of ESD using MLTD in the clinical feasibility evaluation study was limited, and there might be selection bias because inclusion criteria were not defined. A prospective comparative clinical study is needed to demonstrate the utility of the MLTD.

## Conclusions

Using a resected porcine stomach model, we showed that the MLTD increased submucosal dissection ESD speed, not only by experts but also by trainee endoscopists. MLTD also shortened the submucosal dissection time when used by expert endoscopists. The MLTD is easy to attach and extract, and this study shows that the MLTD is potentially an extremely useful device that can reduce the technical difficulty of ESD.

## Supplementary Information

**Additional file 1: Table S1**. Clinical experience of the endoscopists.

**Additional file 2: Video S1.** Endoscopic submucosal dissection using the multi-loop traction device (M-ESD) in an ex vivo porcine stomach model.

## Data Availability

The datasets used and/or analysed during the current study available from the corresponding author on reasonable request.

## Abbreviations

ESD: Endoscopic submucosal dissection
MLTD: Multi-loop traction device
EMR: Endoscopic mucosal resection
GI: Gastrointestinal
