# Supplementary material for: Multi-loop traction device facilitates gastric endoscopic submucosal dissection: ex vivo pilot study and an inaugural clinical experience
Source: BMC Gastroenterol. 2022 Jan 6;22:10. doi: 10.1186/s12876-021-02085-w (PMC8740506; doi:10.1186/s12876-021-02085-w)
Supplement: Supplementary file 1 — Additional file 1: Table S1. Clinical experience of the endoscopists. [file 12876_2021_2085_MOESM1_ESM.docx]

**Supplementary information**

**Supplementary** 1.

Clinical experience of the endoscopists

|  | **EGDs** | **CSs** | **ESDs** |
| --- | --- | --- | --- |
| Expert endoscopist, median (range) | 7000 (3500-10000) | 3000 (1500-10000) | 120 (70-500) |
| Trainee endoscopist, median (range) | 200 (10-600) | 50 (0-300) | 0 (N/A) |

EGD, Esophagogastroduodenoscopy; CS, Colonoscopy; ESD, Endoscopic submucosal dissection
